# Clinical and laboratory findings in COVID-19 adult hospitalized patients from Alborz province / Iran: comparison of rRT-PCR positive and negative

**DOI:** 10.1186/s12879-021-05948-5

**Published:** 2021-03-11

**Authors:** Farnaz Karimi, Amir Abbas Vaezi, Mostafa Qorbani, Fatemeh Moghadasi, Saeed Hassani Gelsfid, Arman Maghoul, Neda Mahmoodi, Zahra Eskandari, Hossein Gholami, Zakiye Mokhames, Mahshid Saleh

**Affiliations:** 1 Department of Pathology, School of Medicine, Alborz University of Medical Sciences, Karaj, Iran; 2 Department of Internal Medicine, School of Medicine, Alborz University of Medical Sciences, Karaj, Iran; 3 Non-Communicable Diseases Research Center, Alborz University of Medical Sciences, Karaj, Iran; 4 Chronic Diseases Research Center, Tehran University of Medical Sciences Endocrinology and Metabolism Research Institute, Tehran, Iran; 5 Student Research Committee, Alborz University of Medical Sciences, Karaj, Iran; 6 Imam Jafar Sadegh hospital, Alborz University of Medical Sciences, Karaj, Iran; 7 Department of Health in emergencies and disasters, Shahid Beheshti University of Medical Sciences, Tehran, Iran; 8 Imam Ali hospital, Alborz University of Medical Sciences, Karaj, Iran; 9 Department of molecular diagnostics, Alborz University of Medical Sciences, Karaj, Iran; 10 Department of Applied Cell Sciences, Faculty of Advanced Technologies in Medicine, Tehran University of Medical Sciences, Tehran, Iran

**Keywords:** rRT-PCR, Laboratory results, COVID19, Clinical findings

## Abstract

**Background:**

The novel coronavirus disease 2019 (COVID-19) was emergency turned into global public health after the first patients were detected in Wuhan, China, in December 2019. The disease rapidly expanded and led to an epidemic throughout China, followed by the rising number of cases worldwide. Given the high prevalence of COVID-19, rapid and accurate diagnostic methods are immediately needed to identify, isolate and treat the patients as soon as possible, decreasing mortality rates and the risk of public contamination by severe acute respiratory syndrome coronavirus 2(SARS-CoV-2).

**Methods:**

This case-control study was conducted in two hospitals in Alborz Province in Iran. All recruited cases in this study were symptomatic adults hospitalized as COVID-19 patients with compatible Computed tomographic (CT) scan findings and available rRT-PCR results. The patients were recruited in this study. The patients were categorized into positive and negative rRT-PCR groups and evaluated for symptoms, initial vital signs, comorbidity, clinical and laboratory findings. Finally, the results were assessed by SPSS software.

**Results:**

Between March 5 to April 5, 2020, 164 symptomatic COVID-19 patients were studied. In total, there were 111 rRT-PCR positive (67.6%) and 53 rRT-PCR negative patients (32.4%). In terms of statistics, the frequency of symptoms revealed no difference, except for cough (P.V:0.008), dizziness (PV: 0.048), and weakness (P.V:0.022). Among initial vital signs, PR (P.V:0.041) and O2 Saturation (PV: 0.014) were statistically different between the two groups. Evaluation of comorbidities revealed no difference except for hyperlipidemia (P.V:0.024). In the comparison of laboratory findings, only WBC count (PV: 0.001), lymphocyte count (PV: 0.001), and Hb (P.V:0.008) were statistically different between the two groups.

**Conclusion:**

In case of the negative rRT-PCR result, it is necessary to take a logical approach, and we recommended that the physician decides according to clinical manifestations, laboratory findings, and positive CT results.

## Background

At the end of 2019, a novel coronavirus was identified as the cause of a cluster of pneumonia cases in Wuhan, a city in China’s Hubei Province. Its rapid spread resulted in an epidemic throughout China, followed by an increasing number of cases in other countries worldwide [1].. Following the detection and announcement of the first cases of COVID-19 on February 20, its high infection and mortality rate has remained ascending [2]. The responsible causative agent, namely SARS-CoV-2, is an enveloped RNA virus of the Coronaviridae family. The transmission of COVID19 occurs via respiratory droplets or contaminated surfaces [3]. It has no specific symptoms. Many cases are asymptomatic, and many others suffer from severe pneumonia with a high incidence of mortality [4]. Most patients present with mild respiratory tract infection, most commonly characterized by fever (82%) and cough (81%). Severe pneumonia and acute respiratory distress syndrome (ARDS) have been reported in 14% of cases with an overall mortality rate of 2% [5]. Nevertheless, these figures are rising concomitant with pandemic expansion depending on the country involved [6]. SARS-CoV infection in humans leads to an acute respiratory illness varying from mild febrile disease to ALI and, in some cases, ARDS and death [7, 8]. Regarding the high rate of infection of COVID-19, Accurate and rapid diagnostic methods are urgently required for detection, isolation, and treatment of patients as soon as possible, which can decrease the community contamination and mortality rate [9]. Computed tomographic (C.T.) imaging is extensively applied for the initial diagnosis of COVID-19, but chest C.T. may not be able to distinguish this disease with other viral causes of pneumonia [10, 11]. In order to confirm the diagnosis of COVID-19, serology tests (IgM and IgG) from throat swabs or blood samples, nucleic acid assay, and gene sequencing have been performed [12]. However, Wuhan clinicians have addressed high false-negative rates in PCR or antibody detection tests [13]. Although rRT-PCR results often take 5 to 6 h to be done, C.T. results can be obtained much faster [9]. Chunqin Long et al. revealed that the sensitivity of C.T. examination was 97.2% at presentation, whereas the sensitivity of the first-round rRT-PCR was 84.6% [9]. This difference may be a function of sample collection because pharyngeal and nasal sampling are more straightforward collection methods, while lower respiratory tract sampling is relatively complicated to perform and bears the risk of infection for susceptible medical staff [14]. The sensitivity of the rRT-PCR kit can also give rise to false-negative results [9].

Simultaneous use of medical history, clinical manifestations, chest C.T., and the viral diagnosis laboratory test has been found to present high sensitivity (92–97%) [15, 16].

A significant challenge for the restriction of SARS-CoV-2 spread is that presymptomatic patients are infectious for others [17]. Recently published reports indicated that patients can be infectious 1–3 days before the onset of symptoms and that up to 40–50% of cases are endangered by asymptomatic or presymptomatic individuals [18, 19]. Patients have high nasopharyngeal loads of virus before or soon after the onset of symptoms, subsequently falling over approximately one week [20]. Patients with severe conditions can release the virus for more extended periods, although the infectious virus’s shedding duration is not precisely known [21]. The severity of the illness determines the evaluation and management of COVID-19. According to initial data taken from China, 81% of people with COVID-19 had mild or moderate disease (people with no pneumonia or mild pneumonia), 14% had severe disease, and 5% showed critical illness [22].

According to the World Health Organization (WHO) guidelines, the risk of COVID-19 infection is not ruled out if one or more negative PCR tests are observed. Factors such as low sample quality (lack of sufficient RNA in the sample), inappropriate time of sampling (delayed or early sampling), improper storage and transportation of the sample, and inherent technical reasons for testing, including virus mutations or PCR inhibitions (such as improper swap use, etc.,) can affect PCR testing and lead to false-negative responses [23]. Due to the rapid spread of COVID19 disease in Iran, an increasing number of patients in a short period, lack of cooperation of some patients in the sampling process, limited access to rRT-PCR testing, assuming rRT-PCR as a time-consuming test, and inability to repeat negative tests, many suspected patients with COVID19 were hospitalized in medical centers based on clinical symptoms, vital signs, laboratory data, and CT-Scan findings.

Due to the limitation of available hospital beds, some of the hospitalized patients show minor clinical symptoms, mild CT-Scan changes, and improved vital signs who had negative rRT-PCR results were discharged by the physician’s clinical judgment a few days of admission. There were several reports of exacerbated symptoms and re-hospitalization of patients in severe conditions. Meanwhile, the positive or negative rRT-PCR results have led the physicians to misinterpret the patient’s clinical conditions. The decision to continue treatment or discharge the patient from the hospital in those with mild clinical symptoms, negative rRT-PCR test, and positive C.T. is an essential question for physicians.

Accordingly, this study investigates the importance of clinical symptoms, initial vital signs, laboratory findings, and chest CT-Scan findings among patients with negative and positive rRT-PCR results.

## Method

This research was a case-control study that was performed in two hospitals in Alborz Province of Iran. The ethics committee approved this study of Alborz University of Medical Sciences with ethics code I.R.ABZUMS.REC 1398.267. Informed consent was taken from subjects, and the inclusion criteria were as follows: all patients with related signs and symptoms of COVID-19 and compatible C.T. findings requiring hospitalization according to National Guideline items (R.R. > 33 or O2 saturation < 93) or physician’s judgment. Exclusion criteria were inconclusive rRT-PCR results, causes of pneumonia other than COVID-19, unclear or incomplete recorded history of patients. Control group: a group of patients with suspected COVID19 with rRT-PCR positive result. Furthermore, laboratory tests such as cell counts, biochemistry, and inflammatory indices were considered an outcome—case group: a group of patients with suspected COVID19 with rRT-PCR negative. Furthermore, laboratory tests such as cell counts, biochemistry, and inflammatory indices were considered as an outcome. Data were collected from the patient’s medical findings and self-reports in determined categories.

### Patients and study design

A total of 164 patients participated in this study from March 5 to April 5, 2020. We divided the patients into two groups: 111 rRT-PCR positive (67.6%) and 53 rRT-PCR negative patients (32.4%). C.T. scans were performed before or at the time of admission for all symptomatic patients, and all C.T. scan results were compatible with COVID − 19. The patients were evaluated for symptoms, initial vital signs, comorbidity, clinical and laboratory findings (CBC, biochemistry parameters, inflammation indices) and date of symptoms’ onset before admission. Real-time reverse transcriptase-polymerase chain reaction (rRT-PCR) was performed on oropharyngeal specimens. In this study, all samples were tested within 48 h after hospitalization, and sampling was done using a standard protocol by trained individuals.

### CT scan

Typical and atypical chest C.T. findings were documented based on C.T. features defined for COVD-19. Radpour et al. designed a low-dose high-resolution computed tomography (HRCT) protocol for the Iranian Society of Radiology to assess patients bearing a high chance of COVID-19 infection. The recommended parameters to diminish the radiation dose were as follows: Kvp: 100–120, mAs: 50–100, pitch: 0.8–1.5, thickness: 1–3 mm, which were used in the present research [24].

### RT-PCR

According to the manufacturer’s instructions, all samples were subjected to RNA extraction with Qiagen Viral Nucleic Acid Kit (QIAcub HT). rRT-PCR was used to detect the presence of SARS-CoV2 using the kits (Molbiol, Germany) provided by WHO targeting the E region for screening and RNA dependent RNA polymerase for confirmation. Invitrogen Superscript III One-Step rRT-PCR System with Platinum Taq DNA Polymerase was used for rRT-PCR (Roche 96 light cycler). For each reaction, 15 μl reaction mix, 1 μl R.T. enzyme, 0.5 μl primer, probe mix, and 3.5 μl PCR grade water were added to 5 μl RNA template. Cycling conditions for amplification of E and RdRP genes were 50 °C for 30 min, 95 °C for 2 min, followed by 45 cycles of 95 °C for 10 s and 60 °C for 30 s (time and temperature are modified) [25]. A cycle threshold value of < 36 Ct was defined as a positive test result. Sensitivity is 5.2 copies per reaction (95% CI: 3.7–9.6) [25].

### Data analysis

We divided all suspected patients into two groups based on rRT-PCR test results (positive and negative). The normal distribution of continuous variables was assessed using the Kolmogorov-Smirnov test. Continuous variables with and without normal distribution were presented as mean (standard deviation (S.D.)) and median (interquartile range (IQR)), respectively. Continuous variables with and without normal distribution were compared using t-test and Mann-Whitney U test across rRT-PCR positive and negative groups, respectively. Categorical variables were presented as numbers (%) and were compared using the Chi-Square test. Multivariate logistic regression (MLR) model was used to distinguish clinical characteristics and laboratory findings between rRT-PCR positive and negative groups. In the MLR model, all variables with a *p*-value less than 0.1 in the univariate model in addition to age and sex were included in the model. MLR was presented as odds ratio (OR) and 95% confidence interval (CI). The SPSS software version 22.0 was used for statistical analysis, and *P* < 0.05 was considered statistically significant.

## Results

### Clinical characteristics of the patients

One hundred sixty-four subjects (44.5% female & 55.5% male) with a mean age of 54 (range: 24–89) years were studied. The result of rRT-PCR for COVID-19 was positive in 111 patients (67.3%) and 53 patients (32.1%) showed negative rRT-PCR results. Positive and negative rRT-PCR results are shown based on gender (P.V = 0.384) and age (P.V = 0.38) distribution in Table 1.

**Table 1:** Clinical characteristics and initial vital signs of the patients in rRT-PCR positive and negative groups

Variables	Total	PCR Negative	PCR Positive	*P*-Value
**Gender NO%**				
Female	73 (44.5)	21 (40.0%)	52 (46.8%)	0.384
Male	91 (55.5)	32 (60.0%)	59 (53.2%)	0.384
**Age. Mean (SD)**				
Age	54.0	52.6(16.7)	54.8(14.3)	0.38
**Initial vital sign. Mean (SD)**				
RR breaths/min	19.27	19.1(3.4)	19.3(2.5)	0.384
Temp	37.0	36.8(1.0)	37.1(0.8)	0.207
Temp> 37.8	38(23.2)	10(18.9)	28(25.2)	0.367
PR beats/min	99.1	98.6(23.0)	99.4(17.2)	0.041
Sys BP, mm Hg	130.2	129.8(22.0)	130.3(19.3)	0.814
Dias BP, mm Hg	79.3	78.7(11.6)	79.5(13.2)	0.770
O2 Sat	91.7	89.5(8.0)	91.8(5.5)	0.014
O2 Sat < 90%	37(22.6)	20(37.7)	17(15.3)	< 0.001
**Symptoms.NO(%)**				
Cough	118(72.0)	31(58.5)	87(78.4)	0.008
Dyspnea	94(57.3)	29(54.7)	65(58.6)	0.642
Sore throat	30(18.3)	9(17.0)	21(18.9)	0.764
Fever	83(50.6)	23(43.4)	60(54.1)	0.202
Chills	60(36.6)	15(28.3)	45(40.5)	0.128
Headache	51(31.1)	13(24.5)	38(34.2)	0.209
Dizziness	37(22.6)	7(13.2)	30(27.0)	0.048
Weakness	80(48.8)	19(35.8)	61(55.0)	0.022
Muscular pain	75(45.7)	23(43.4)	52(46.8)	0.678
Diarrhea	27(16.5)	11(20.8)	16(14.4)	0.306
Abdominal pain	14(8.5)	5(9.4)	9(8.1)	0.776
Anorexia	73(44.5)	18(34.0)	55(49.5)	0.060
Nausea	52(31.7)	14(26.4)	38(34.2)	0.314
Vomiting	30(18.3)	7(13.2)	23(20.7)	0.244
**Comorbidity.NO(%)**				
Diabetes	39(23.8)	12(22.6)	27(24.3)	0.813
Hyperlipidemia	10(6.1)	0(0.0)	10(9.0)	0.024
Hypertension	39(23.8)	11(20.8)	28(25.2)	0.529
CHD	24(14.6)	8(15.1)	16(14.4)	0.908
CKD	1(0.6)	0(0.0)	1(0.9)	0.501
Asthma	11(6.7)	5(9.4)	6(5.4)	0.335
COPD	0(0.0)	0(0.0)	0(0.0)	_
Cirrhosis	0(0.0)	0(0.0)	0(0.0)	_
Autoimmune disease	1(0.6)	0(0.0)	1(0.9)	0.488
History of malignancy	1(0.6)	1(1.9)	0(0.0)	0.147
Recent chemoradiotherapy	1(0.6)	1(1.9)	0(0.0)	0.147
Current steroid use	1(0.6)	0(0.0)	1(0.9)	0.488
Immunosuppressant drug use	1(0.6)	0(0.0)	1(0.9)	0.488

*RR* respiratory rate, *Temp* temperature, *PR* pulse rate, *Sys BP* systolic blood pressure, *Dias BP* diastolic blood pressure, *O2 Sat* O2 saturation, *CHD* chronic heart disease, *CKD* chronic kidney disease, *COPD* chronic obstructive pulmonary disease

Comparing the symptoms in positive and negative rRT-PCR groups showed no significant difference except for dizziness (*p* = 0.048), cough (*p* = 0.008), and weakness (*p* = 0.022), which were slightly more frequent in the positive rRT-PCR group than the negative rRT-PCR group.

Cough (31, 58.5%), dyspnea (93, 57.1%), fever (60, 54.1%) and weakness (61, 55.0%) were the most common symptoms in positive RT-PCR group. In negative rRT-PCR group, cough (87, 78.4%), dyspnea (29, 54.7%), fever (23, 43.4%) and muscular pain (23, 43.4) were the most prevalent symptoms. Statistical analysis of initial vital signs showed that R.R., Temp, SBP, and DBP were similar between positive and negative rRT-PCR groups, while pulse rate P.R. (H.R.) (*p* < 0.041) and O2 saturation (SO2) (*p* < 0.014) were significantly different in the two groups, which are shown in Table 1. In a positive rRT-PCR group, the average (mean ± SD) of P.R. (H.R.) and SO2 were 99.4 (17.2) and 91.8 (5.5), respectively, which were 98.6 (23.0) and 89.5 (8.0) in the negative rRT-PCR group.

According to the data, the average P.R. was slightly higher in the positive rRT-PCR group, and the average of SO2 in the negative rRT-PCR group was slightly lower.

### Clinical findings

The two groups had significant similarities concerning comorbidities, and only hyperlipidemia was significantly higher in the positive rRT-PCR group (10, 9.0%) (*p* = 0.024). The most common comorbidities in the positive rRT-PCR group were hypertension and diabetes (28, 25.2%) and (27, 24.3%), respectively. Also, in the negative rRT-PCR group, hypertension (11, 20.8%) and diabetes (12, 22.6%) were the most common comorbidities (Table 1).

We also analyzed the appearance date of symptoms before admission and found no significant difference between groups (Table 2).

**Table 2:** Laboratory findings of patients infected with 2019-nCoV on admission to hospital in rRT-PCR Positive and rRT-PCR Negative groups

Variable(n/N)	Total	PCR Negative(*n* = 53)	PCR Positive(*n* = 111)	*P* value
^a^Hb mg/dl	13.5	13.1(2.4)	13.7(1.6)	0.008
WBC count, × 10^9^/L	5800(4400–7700)	6850.0(5137.5–8587.5)	5410.0(4280.0–6800.0)	0.001
Lymph count, × 10^9^/L	1.2(0.9–1.6)	1.5(0.9–2.1)	1.1(0.8–1.4)	0.001
Lymph count< 1.1 × 10^9^/L	68/164(41.7)	16/53(30.8)	52/111(46.8)	0.05
Platelet count, × 10^9^/L	194.5(136.5–249.2)	212.0(144.0–263.0)	191.0(135.0–247.0)	0.363
ESR(124/164)	46(30.2–65.7)	50.0(33.0–68.0)	45.0(30.0–65.0)	0.625
CRP mg/dl (159/164)	39.7(11.0–74.0)	36.5(8.5–57.7)	42.7(13.0–80.1)	0.180
CRP > 6 mg/dl	123/159(80.5)	40/50(80.0)	88/109(80.7)	0.914
LDH, U/L (104/164)	472(362.2–591.5)	414.0(297.0–557.7)	484.0(297.0–557.7)	0.195
LDH > 245	98/104(94.2)	27/30(90.0)	71/74(95.9)	0.239
AST, U/L(80/164)	39(30.0–48.0)	35.5(30.0–48.0)	40.0(30.2–48.7)	0.418
ALT, U/L (80/164)	33(24.0–41.0)	33.0(22.0–57.0)	34.0(24.5–41.0)	0.983
CPK U/L(23/164)	159(51.0–236.0)	93.0(45.0–236.0)	174.5(61.9–292.7)	0.671
Sodium mmol/L (130/164)	136(133–138)	136.0(133.0–138.0)	135.0(133.0–138.0)	0.445
Potassium mmol/L (130/164)	4.1(3.8–4.4)	4.1(3.9–4.5)	4.0(3.8–4.3)	0.067
Magnesium mmol/L (60/164)	2.0(1.9–2.2)	2.1(1.9–2.2)	2.0(1.9–2.3)	0.726
BUN mg/dl(158/164)	12.1(9.0–16.7)	12.0(9.0–18.1)	12.1(9.0–16.2)	0.455
Cr mmol/L (158/164)	1.0(0.9–1.2)	1.0(0.8–1.1)	1.0(0.9–1.2)	0.151
Date of symptoms before admission	6.5(4.0–8.7)	6.0(3.0–9.0)	7.0(4.0–8.0)	0.958

Other data are Median (IQR), or n/N (%)

*Hb* hemoglobin, *WBC* white blood cell, *Lymph* lymphocyte, *ESR* erythrocyte sedimentation rate, *CRP* C-reactive protein, *LDH* lactate dehydrogenase, *AST* aspartate aminotransferase, *ALT* alanine aminotransferase, *CPK* creatine phosphokinase, *BUN* blood urea nitrogen, *Cr* Creatinine

^a^This data is Mean (SD)

### Laboratory parameters

Table 2 compares the laboratory parameters of patients with positive and negative rRT-PCR results. There were many similarities in laboratory findings between positive and negative rRT-PCR groups, among which WBC (*p* = 0.001), lymphocyte count (p = 0.001), and Hb (*p* = 0.008) were statistically significant.

In the positive rRT-PCR group, white blood cell median (IQR) was 5.4 (4.2–6.8), lymphocyte count median (IQR) was 1.1 (0.879–1.4), and Hb median was 13.7 (1.6). In a negative rRT-PCR group, the following values were as follows: white blood cell median (IQR) 5.4 (4.2–6.8), lymphocyte count median (IQR) 1.1(0.8–1.4), and Hb (mean ± SD) 13.7 (1.6).

In the multivariate model, all variables with a *p*-value less than 0.1 in the univariate model (Tables 1 and 2) were included in the model. In the MLR model, among all clinical characteristics and laboratory findings only presence of cough symptom (OR: 2.74; 95% CI: 1.11–6.8; *p*-value: 0.02) and increasing Hb (OR: 1.26; 95% CI: 1.02–1.57; *p*-value:0.03) was associated with rRT-PCR positivity.

## Discussion

The pandemic of novel coronavirus 2019 (COVID-19) has been a matter of international concern due to the fast spread of the disease [26]. During the first phase of the COVID-19 outbreak, the diagnosis of the disease was complicated due to the diversity of symptoms and imaging results and the severity of the disease at the time of presentation [4]. Currently, the RT-PCR amplification of viral RNA is considered as a “gold standard” method. However, initial RT-PCR is not always positive in patients with COVID-19 infection [16, 27]. In this case, chest C.T. images could play an essential role in detecting pulmonary parenchyma lesions in the patients suspected of COVID-19 infection.

Nevertheless, it does not mean that the abnormalities of C.T. images could be observed in COVID-19 infection while the initial RT-PCR is positive or negative [16, 27, 28]. Previously published studies have suggested that in some COVID-19 patients, a false-negative rRT-PCR result be observed [29, 30]. False-negative results may be caused by various factors such as human errors when following the diagnostic kit protocol, the sensitivity of reagents, the site and method of specimen sampling, and collection times [31].

In Yang et al.’s study, the total positive rate of RT-PCR for throat swab samples was reported to be about 30–60% at initial presentation despite limitations of sample collection, transportation, and kit performance. In this study, all patients were evaluated for clinical manifestations and radiological examination [32]. One of the studies in Wuhan revealed that a considerable ratio of COVID-19 patients may have had an initial negative result for rRT-PCR test and that the Positively diagnosed patients had a higher tendency to turn into more serious/ severe cases. This study stated that patients with negative rRT-PCR who presented with typical clinical manifestations should not be ignored and the PCR test should be repeated for them [33].

Our study also examined the date of symptoms’ onset before admission to the hospital, and no significant differences were observed between rRT-PCR positive and rRT-PCR negative groups. Yang et al. revealed that the sputum sample collected during 8–14 days showed a higher positive qPCR rate than the nasal and throat swabs samples in both severe and mild cases. The positive qPCR rate of throat samples decreased a few days after the onset of symptoms to hospitalization and performing PCR tests. The likelihood of a positive throat samples test and symptoms decreased after 15 days [32].

In our study, patients were hospitalized based on clinical manifestations, laboratory test results, and a positive C.T. scan corresponding to COVID-19, and the rRT-PCR test result was ready after 48 h. We performed rRT-PCR on oropharyngeal specimens. In this research, we found 68.1% rRT-PCR positive results, a percentage that may be due to the same test conditions, including the operator performing the test, sampling method, diagnostic kit, etc. for all samples, we detected a slight difference between positive and negative rRT-PCR patients in terms of clinical and laboratory findings, initial vital signs and comorbidity. Therefore, patients with negative rRT-PCR should not be discharged from the hospital, especially when presenting similar clinical manifestations to positive rRT-PCR patients.

This study has several limitations. First, it was impossible to repeat negative rRT-PCR tests due to shortcomings such as the lack of laboratory testing capacity, insufficient staff, and limited diagnostic kits. Second, rRT-PCR tests were performed only on hospitalized patients and did not assess COVID-19 suspects who had been recommended home quarantine and rest. Third, the physicians judged the patient’s hospitalization requirement based on clinical symptoms and lung C.T. scan because it was impossible to perform an rRT-PCR test at first. Therefore, we had no patients with negative C.T. scans and positive rRT-PCR. Fourth, incomplete medical records of a few patients due to the high number of patients’ admission to the hospital emergency ward, insufficient number of physicians and nurses to complete the history, and the patients’ inability to express their history were another limitation of the present research.

## Conclusion

At the time of this study, our physician’s most common trend was to discharge suspected COVID19 PCR negatives patients in the earlier phases. Also, considering limited sources for the PCR test that was not feasible to recheck the PCR test in most patients; this prejudgment was common to underestimate these kinds of patients clinically. We observed many initially discharged patients returning to our centers with more clinical symptoms and impaired lab tests. Finally, we conclude that the decision on COVID-19 patients should not exclusively depend on rRT-PCR positivity during the pandemic. Clinical manifestations, laboratory findings, and positive C.T. results play a critical role in clinicians’ decisions, especially in countries with a high prevalence of COVID-19 with lower medical facilities.

The patients would be isolated from other healthy individuals and not discharged from the hospital until they have fully recovered. We suggest that it would be better to investigate many people to obtain more accurate results. This study compared clinical characteristics, initial vital signs, and laboratory data of early stage of COVID-19 in rRT-PCR positive and negative groups in Iran, which has an absolute value for future control and research. The patients’ follow-up and outcome evaluation between rRT-PCR positive and negative groups could be the subjects for further research.

## Data Availability

The datasets used and/or analyzed during the current study are available from the corresponding author on reasonable request.

## Abbreviations

COVID-19: Coronavirus disease 2019
SARS-CoV-2: Severe acute respiratory syndrome coronavirus2
rRT-PCR: Real-time reverse transcription polymerase chain reaction
RNA: Ribonucleic acid
ALI: Acute lung injury
ARDS: Acute respiratory distress syndrome
CT: Computed tomographic
WHO: World Health Organization
HRCT: High resolution computed tomography
IQR: Interquartile range
